# Honokiol induces apoptosis-like death in *Cryptocaryon irritans* Tomont

**DOI:** 10.1186/s13071-023-05910-1

**Published:** 2023-08-16

**Authors:** Zi-Chen Zhao, Man-Yi Jiang, Ji-Hui Huang, Chuan Lin, Wei-Liang Guo, Zhi-Hong Zhong, Qing-Qin Huang, Shao-Long Liu, Heng-Wei Deng, Yong-Can Zhou

**Affiliations:** 1 Hainan Provincial Key Laboratory for Tropical Hydrobiology and Biotechnology, College of Marine Science, Hainan University, Haikou, 570228 People’s Republic of China; 2 School of Life Sciences, Hainan University, Haikou, 570228 People’s Republic of China; 3 Aquaculture Department, Hainan Agriculture School, Haikou, 571101 People’s Republic of China; 4 Technology Center of Haikou Customs District, Haikou, 570105 People’s Republic of China

**Keywords:** *Cryptocaryon irritans*, Honokiol, Apoptosis-like death, Endoplasmic reticulum stress

## Abstract

**Background:**

*Cryptocaryon irritans*, a common parasite in tropical and subtropical marine teleost fish, has caused serious harm to the marine aquaculture industry. Honokiol was proven to induce *C. irritans* tomont cytoplasm shrinkage and death in our previous study, but the mechanism by which it works remains unknown.

**Methods:**

In this study, the changes of apoptotic morphology and apoptotic ratio were detected by microscopic observation and AnnexinV-FITC/PI staining. The effects of honokiol on intracellular calcium ([Ca^2+^]_i_) concentration, mitochondrial membrane potential (Δ*Ψ*m), reactive oxygen species (ROS), quantity of DNA fragmentations (QDF) and caspase activities were detected by Fluo-3 staining, JC-1 staining, DCFH-DA staining, Tunel method and caspase activity assay kit. The effects of honokiol on mRNA expression levels of 61 apoptosis-related genes in tomonts of *C. irritans* were detected by real-time PCR.

**Results:**

The results of the study on the effects of honokiol concentration on *C. irritans* tomont apoptosis-like death showed that the highest levels of prophase apoptosis-like death rate (PADR), [Ca^2+^]_i_ concentration, ROS, the activities of caspase-3/9 and the lowest necrosis ratio (NER) were obtained at a concentration of 1 μg/ml, which was considered the most suitable for inducing *C. irritans* tomont apoptosis-like death. When *C. irritans* tomonts were treated with 1 μg/ml honokiol, the [Ca^2+^]_i_ concentration began to increase significantly at 1 h. Following this, the ROS, QDF and activities of caspase-3/9 began to increase significantly, and the Δ*Ψ*m began to decrease significantly at 2 h; the highest PADR was obtained at 4 h. The mRNA expression of 14 genes was significantly upregulated during honokiol treatment. Of these genes, *itpr2*, *capn1*, *mc*, *actg1*, *actb*, *parp2*, *traf2* and *fos* were enriched in the pathway related to apoptosis induced by endoplasmic reticulum (ER) stress.

**Conclusions:**

This article shows that honokiol can induce *C. irritans* tomont apoptosis-like death. These results suggest that honokiol may disrupt [Ca^2+^]_i_ homeostasis in ER and then induce *C. irritans* tomont apoptosis-like death by caspase cascade or mitochondrial pathway, which might represent a novel therapeutic intervention for *C. irritans* infection.

**Graphical abstract:**

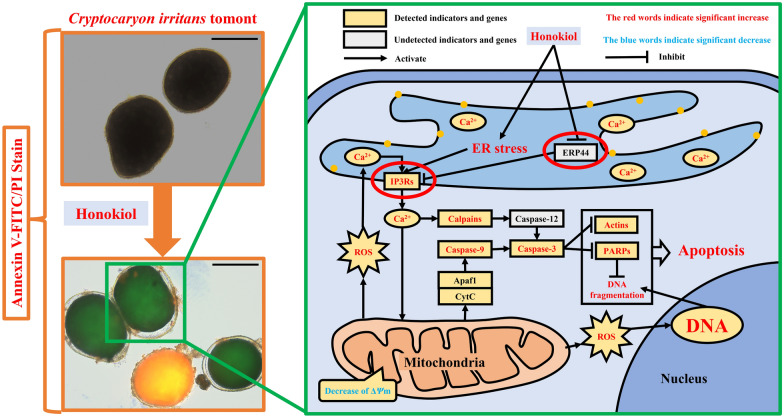

**Supplementary Information:**

The online version contains supplementary material available at 10.1186/s13071-023-05910-1.

## Background

*Cryptocaryon irritans*, a common protozoan parasite of marine teleost fish, causes “white spot” disease [1]. This disease is mainly prevalent in tropical and subtropical sea areas [2–4]. Its life cycle includes four stages: trophont, protomont, tomont, and theront [5]. Tomont is the longest-lasting, free-living stage of *C. irritans*. Tomonts have strong resistance to drugs and harsh environments because of their hard cysts. Tomonts can still produce infectious theronts after being preserved at 12 °C for 3 months [5]. It is difficult to completely remove *C. irritans* tomonts in an open mariculture environment, which makes the prevention and control of white spot disease very difficult. It is a good strategy to prevent and treat parasites by inducing their spontaneous death; this has the advantages of low probability of drug resistance and host inflammation. It is well known that apoptosis is a highly regulated process of cell death [6]. In recent years, apoptosis has provided a new treatment for many diseases, such as inflammation, cancer, leishmaniasis, malaria, and toxoplasmosis [7–14]. The apoptosis-like death pathway has also been found in many protozoa, such as Leishmania, *Plasmodium falciparum*, *Tetrahymena thermophila*, *Trypanosoma cruzi*, *Blastocystis hominis*, *Toxoplasma gondii*, and *Ichthyophthirius multifiliis* [12–21], providing a new way to treat parasitic diseases. Many apoptosis-related genes of *C. irritans* have been found via transcriptome analysis [22–26], which indicates that *C. irritans* might have an apoptosis-like death pathway. Honokiol, one of the main active components of *Magnolia officinalis*, has been reported to induce apoptosis of cancer cells and *Candida albicans* via the endoplasmic reticulum (ER) stress pathway [27–30]. Our previous studies demonstrated that honokiol significantly inhibited the proliferation and hatching of *C. irritans* tomonts. The resulting *C. irritans* tomont cytoplasm obviously shrank without cytoplasm or cell membrane damage [31], indicating that honokiol might induce *C. irritans* tomont apoptosis-like death. However, further experiments are needed to confirm this speculation, and the mechanism remains to be uncovered.

In this article, Annexin V-FITC/PI staining was used to determine whether honokiol induces *C. irritans* tomont apoptosis-like death. The effects of honokiol at various treating concentrations and times on the morphologies, normal rate (NOR), prophase apoptosis-like death rate (PADR), anaphase apoptosis-like death rate (AADR), necrosis rate (NER), intracellular calcium concentration ([Ca^2+^]_i_ concentration), mitochondrial membrane potential (Δ*Ψ*m), reactive oxygen species (ROS), quantity of DNA fragmentations (QDF), caspase activities, and mRNA expression of apoptosis-related genes of *C. irritans* tomont were investigated to uncover the mechanism of honokiol for inducing *C. irritans* tomont apoptosis-like death.

## Methods

### Fish, drugs, and parasite

*Trachinotus ovatus*, weighing 80.0 ± 10.6 g, were purchased from a marine cage culture, Lingshui County, Hainan Province, P.R. China. The fish were soaked in seawater containing 1 ml/l formaldehyde for 10 min and acclimated in 2000 l aquaria for 21 days (diameter = 2 m, height = 1 m) equipped with aerators and a flow-through water system (water depth 0.6 m, water temperature 29.0 ± 2.0 ℃, pH 7.8 ± 0.2, salinity 30 ± 0.5, dissolved oxygen [DO] 6.8 ± 0.1 mg/l). The fish were fed marine fish feed (Zhongshan President Enterprises Co., Ltd., P.R. China) at 4% body weight three times (9:00, 16:00, and 22:00) every day.

Honokiol with a high-performance liquid chromatography (HPLC) purity ≥ 98% was purchased from Century Aoke Biological Technology Co., Ltd. (P.R. China). An amount of 250 mg of honokiol was dissolved in 25 ml 50% (v/v) ethanol aqueous solution and diluted to 800, 400, 200, 100, 60, and 0 (control sample) μg/ml with 50% (v/v) ethanol aqueous solution.

*Cryptocaryon irritans* used in this study were isolated from *T. ovatus* with white spot disease, which were obtained from a marine cage culture in Lingao County, Hainan Province, P.R. China, and passaged and propagated in *T. ovatus* [31]. All *C. irritans* tomonts used in this study were newly collected between 8:00 a.m. and 9:00 a.m. on the day of the experiment.

### Experiment design

*Cryptocaryon irritans* tomonts were collected, resuspended in 1 ml filter-sterilized seawater, and added to the assigned wells in a 24-well microplate. Then, 10 μl of 0 (control sample), 60, 100, 200, 400, and 800 μg/ml honokiol 50% (v/v) ethanol aqueous solutions were added to the assigned wells in the 24-well microplates, respectively. The final honokiol concentrations were 0.0 (control sample), 0.6, 1.0, 2.0, 4.0, and 8.0 μg/ml, respectively. The 24-well microplates were placed in a light incubator (GZX250E, Tianjin Taisite Instrument Co., Ltd., P.R. China) at 27 ± 0.5 °C for 8 h. The morphologies, NOR, PADR, AADR, NER, [Ca^2+^]_i_ concentration, Δ*Ψ*m, ROS, QDF, and caspase activities of the above-treated *C. irritans* tomonts were analyzed using a direct microscopic observation method, an Annexin V-FITC/PI apoptosis detection kit, a Fluo-3 AM calcium concentration detection kit, a mitochondrial membrane potential assay kit, a reactive oxygen species assay kit, a terminal deoxynucleotidyl transferase dUTP nick end labeling (TUNEL) apoptosis assay kit, and a caspase-3/8/9 activity assay kit, respectively. *Cryptocaryon irritans* tomonts were treated with honokiol at the optimum concentration according to the results of the above experiments for 0, 1, 2, 4, 8, and 16 h. Their morphologies, NOR, PADR, AADR, NER, [Ca^2+^]_i_ concentration, Δ*Ψ*m, ROS, QDF, and caspase activities were also analyzed using the same methods as mentioned above. *Cryptocaryon irritans* tomonts were treated with honokiol at the conditions with the highest apoptosis rate of tomonts according to the results of the above experiments, and then the inhibition rate of tomonts hatching was observed according to the method described by Zhong et al. [31]. Each well assigned to caspase activity analysis and the mRNA expression of apoptosis-related genes analysis contained 1000 *C. irritans* tomonts, while each well assigned to the other analyses contained 100 *C. irritans* tomonts. Each analysis was carried out five times in parallel.

### Morphology observation and Annexin V-FITC/PI stain

The NOR, PADR, AADR, and NER of *C. irritans* tomonts were analyzed using an Annexin V-FITC/PI apoptosis detection kit (Beijing Solarbio Science & Technology Co., Ltd., P.R. China). The *C. irritans* tomonts, treated as described in “Experiment Design,” were washed twice with 1 ml ice-cold PBS (pH = 7.4, 0.01 mol/l) and resuspended in 100 μl binding buffer. A solution of 5 μl Annexin V-FITC was added, and the tomonts were incubated at 27 ± 0.5 ℃ in the dark for 10 min. Then, a solution of 5 μl PI was added, and the tomonts were incubated at 27 ± 0.5 ℃ in the dark for 5 min, washed twice with 1 ml ice-cold PBS, and transferred to 384-well microplates (each well contained a *C. irritans* tomont). A total of 150 *C. irritans* tomonts were analyzed in parallel with a fluorescence microplate reader (BioTek Synergy H1, BioTek Instruments, Inc., USA) at Ex/Em = 488/525 nm and Ex/Em = 488/630 nm, and their micromorphologies were observed under a fluorescence inversion microscope (DMi8 + DFC7000T, Leica Microsystems, Germany). Four-quadrant apoptosis diagrams were drawn using a logarithm transformed Annexin V-FITC fluorescence intensity [LG (*A*_Ex/Em = 488/525 nm_)] as the abscissa axis and a logarithm transformed PI fluorescence intensity [LG (*A*_Ex/Em = 488/630 nm_)] as the ordinate axis. Normal (in the I quadrant), prophase apoptosis-like death (in the II quadrant), anaphase apoptosis-like death (in the III quadrant), and necrosis (in the IV quadrant) *C. irritans* tomonts were identified according to the four-quadrant apoptosis diagrams, and their numbers were recorded [28]. Finally, the percentages of the NOR, PADR, AADR, NER, and *C. irritans* tomonts were calculated.

### [Ca^2+^]_i_ concentration determination

The [Ca^2+^]_i_ concentrations in *C. irritans* tomonts were determined using a Fluo-3 AM calcium concentration detection kit (Beijing Solarbio Science & Technology Co., Ltd., P.R. China). The *C. irritans* tomonts, treated as described in “Experiment Design,” were washed twice with 1 ml PBS (pH = 7.4, 0.01 mol/l) and incubated in 200 μl Fluo-3 AM reaction mix (5 µmol/l) at 27 ± 0.5 ℃ in the dark for 20 min. Then, 1 ml 1% fetal bovine serum-Hank’s balanced salt solution (HBSS, pH = 7.4, 0.01 mol/l) was added, and the tomonts were incubated at 27 ± 0.5 ℃ in the dark for 40 min, washed twice with 1 ml 2-[4-(2-hydroxyethyl)-1-piperazinyl] ethanesulfonic acid buffer (HEPES, pH = 7.4, 0.01 mol/l), and resuspended in 100 μl HEPES at 27 ± 0.5 ℃ in the dark for 10 min. The fluorescence intensity was determined using a fluorescence microplate reader (BioTek Synergy H1, BioTek Instruments, Inc., USA) at Ex/Em = 488/525 nm. The results were expressed as the total fluorescence intensity per 100 *C. irritans* tomonts.

### Δ*Ψ*m determination

The Δ*Ψ*m in *C. irritans* tomonts was determined using a mitochondrial membrane potential assay kit with JC-1 (Beijing Solarbio Science & Technology Co., Ltd., P.R. China). The *C. irritans* tomonts were treated with carbonyl cyanide 3-chlorophenylhydrazone (CCCP, 10 μmol/l) for 20 min as a positive control. The *C. irritans* tomonts, treated as described in “Experiment Design” and with CCCP, were washed twice in 1 ml PBS (pH = 7.4, 0.01 mol/l), incubated in 10 µg/ml JC-1 reaction mix for 20 min at 27 ± 0.5 ℃ in the dark, washed twice with 1 ml JC-1 buffer solution (1 ×), and resuspended in 100 μl JC-1 binding buffer (1 ×). The fluorescence intensity was determined using a fluorescence microplate reader (BioTek Synergy H1, BioTek Instruments, Inc., USA) at Ex/Em = 490/530 nm and Ex/Em = 525/590 nm. The results were expressed as the rate of mitochondrial membrane potential polymer to monomer (polymer/monomer) per 100 *C. irritans* tomonts [32].

### ROS determination

The ROS accumulation in *C. irritans* tomonts was determined by a reactive oxygen species assay kit (Beijing Solarbio Science & Technology Co., Ltd., P.R. China). The *C. irritans* tomonts, treated as described in “Experiment Design,” were washed twice in 1 ml PBS (pH = 7.4, 0.01 mol/l), incubated in 200 μl 2',7'-dichlorodihydrofluorescein diacetate (DCFH-DA, 10 μmol/l) reaction mix at 27 ± 0.5 ℃ in the dark for 20 min, washed twice in 1 ml PBS (pH = 7.4, 0.01 mol/l), and resuspended in 100 μl PBS (pH = 7.4, 0.01 mol/l). The fluorescence intensity was determined using a fluorescence microplate reader (BioTek Synergy H1, BioTek Instruments, Inc., USA) at Ex/Em = 488/525 nm. The results were expressed as the total fluorescence intensity per 100 *C. irritans* tomonts.

### TUNEL assay

The QDF in *C. irritans* tomonts was determined using a TUNEL apoptosis assay kit (Beijing Solarbio Science & Technology Co., Ltd., P.R. China). The *C. irritans* tomonts, treated as described in “Experiment Design,” were washed twice in 1 ml PBS (pH = 7.4, 0.01 mol/l), fixed in 200 μl 4% (w/v) paraformaldehyde at room temperature for 30 min, washed three times in 1 ml PBS (pH = 7.4, 0.01 mol/l), permeabilized with 200 μl 0.01% Triton-PBS (pH = 7.4, 0.01 mol/l) in ice for 2 min, washed twice in 1 ml PBS (pH = 7.4, 0.01 mol/l), and incubated in 100 μl TUNEL reaction mix (1 ×) at 27 ± 0.5 ℃ in the dark for 60 min, washed twice in 1 ml PBS (pH = 7.4, 0.01 mol/l), and resuspended in 100 μl PBS (pH = 7.4, 0.01 mol/l). The fluorescence intensity was determined using a fluorescence microplate reader (BioTek Synergy H1, BioTek Instruments, Inc., USA) at Ex/Em = 550/590 nm. The results were expressed as the total fluorescence intensity per 100 *C. irritans* tomonts.

### Caspase activity determination

The activities of caspases, including caspase-3/8/9 in the *C. irritans* tomonts, were respectively determined using a caspase-3/8/9 activity assay kit (Beijing Solarbio Science & Technology Co., Ltd., P.R. China). On ice, the *C. irritans* tomonts, treated as described in “Experiment Design,” were washed twice in 1 ml ice-cold PBS (pH = 7.4, 0.01 mol/l), a lysis buffer was added at a ratio of 1: 10 (*C. irritans* tomonts mass: lysis buffer, g:ml), ground to homogenate on the ice using a grinding rod, cracked in ice for 15 min, and centrifugated (15000 g, 4 ℃, 15 min). The supernatants were then collected. The supernatants, caspase reaction buffer, and substrates (Asp-Glu-Val-Asp-p-nitroanilide for caspase-3 activity determination, N-acetyl-Ile-Glu-Thr-Asp-p-nitroanilide for caspase-8 activity determination, and Leu-Glu-His-Asp-p-nitroanilide for caspase-9 activity determination) were added to 96-well enzyme plates and incubated at 37 ℃ for 8 h, determined by microplate reader (BioTek Synergy H1, BioTek Instruments, Inc., USA) at 405 nm. The activities of caspase-3/8/9 were calculated using the following function:

$$\mathrm{Caspase activity }\left(U/mg Protein\right)=\frac{X\times {V}_{1}}{{V}_{2}\times {C}_{protein}\times T}$$

where *X* is the result of the calculation of the absorbance at 405 nm in the caspase standard curve (μmol/l), *V*_1_ is the total volume of the reaction system (μl), *V*_2_ is the volume of the added supernatant (μl), *T* is the reaction time (h), and *C*_*protein*_ is the total protein concentration in the supernatant (mg/ml) determined by the Bradford method [33].

### Effects of honokiol on the mRNA expression of apoptosis-related genes

The *C*. *irritans* tomonts were treated with honokiol at the optimum concentration obtained according to the results of experiment design for 0, 1, 2, 4, 8, and 16 h, and the mRNA expression of the 61 apoptosis-related genes obtained by blast according to the *C. irritans* genome (SRX12890364, SRX12890363) [34] was analyzed using the RT-PCR method. Each well assigned to mRNA expression of apoptosis-related gene analysis contained 1000 *C. irritans* tomonts. Each analysis was carried out in triplicate. The 61 apoptosis-related genes and their primers designed by the Primer 3 plus software (https://www.primer3plus.com/) according to the primer design rules [70] are listed in Additional file 1: Table S1. The specificities of the primers used in this study were confirmed using BLAST tool (https://blast.ncbi.nlm.nih.gov/Blast.cgi). Eastep Super Total RNA Extraction LS1040 (Promega Corp., USA) was used according to the manufacturer's instructions to extract total RNA by the glass tissue homogenizer from the treated *C. irritans* tomonts, and DNase I (Promega Corp., USA) was used to digest the contaminating DNA. Then, agarose gel electrophoresis and an ultramicro spectrophotometer (NanoPhotometer NP80, IMPLEN GMBH, Germany) were used to ensure the quality of RNA, and the cDNA sequence was synthesized using Eastep RT Master MIX Kit LS2054 (Promega Corp., USA). The mRNA expressions were detected using the CFX96 Real-Time PCR Detection System (Bio-Rad, Hercules, CA, USA) with a 10 μl reaction mixture containing 5 μl (2 ×) Eastep qPCR Master Mix (Vazyme Biotech Co., Ltd., P.R. China), 1.0 μl cDNA template, 0.2 μl each primer (10 μmol/l), and 3.6 μl nuclease-free water. Each sample was tested in triplicate. The real-time PCR cycling conditions were as follows: 120 s 95 °C initial denaturation, 40 cycles of 15 s 95 °C denaturation, and 60 s 60 °C annealing/extension. Dissociation curve analysis (65 to 95 °C: increment 0.5 °C for 5 s) was performed to verify the amplification of a single product. The *C. irritans* 18S rRNA gene sequence (JN636814.1) was used as the internal reference gene. Furthermore, the specificities of the primers were confirmed by the fluorescence quantitative melting curve used the CFX96 Real-Time PCR Detection System (Bio-Rad, Hercules, CA, USA). After real-time PCR, the mRNA expression of the apoptosis-related genes was calculated u the method of 2^−△△Ct^.

### Statistical analysis

All the experimental indexes were tested using Tukey’s test with SPSS 25.0 (IBM Corp., USA) to determine the significant difference between the experimental sample and the control sample. A difference was considered significant when the* p* values were < 0.05 and extremely significant when the *p* values were < 0.01. The chart was plotted using OriginPro 9.0 (OriginLab Corp., USA), and the data were expressed as mean ± standard deviation (SD).

## Results

### Results of morphology observation and Annexin V-FITC/PI stain

**Fig. 1:**
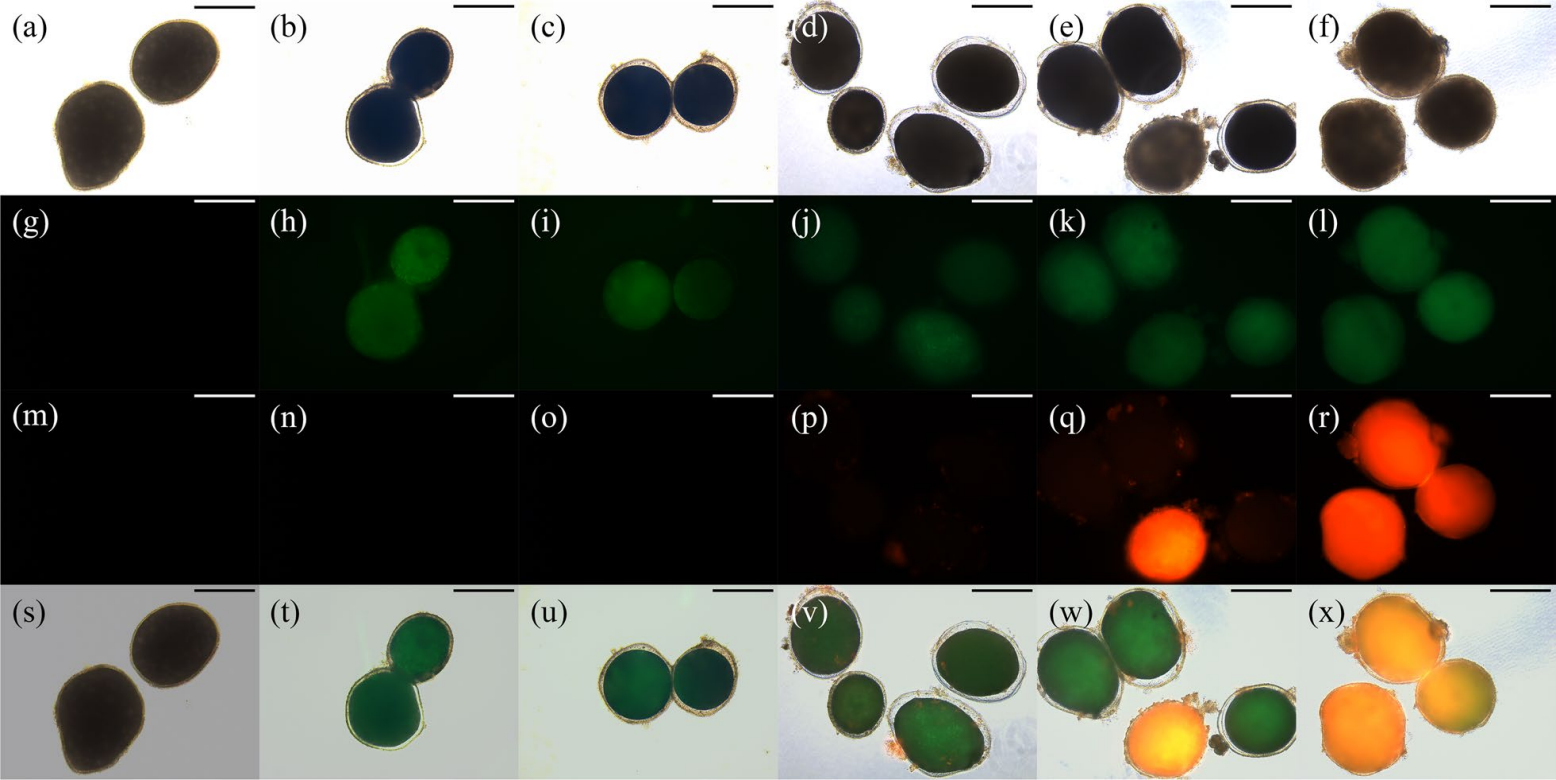
Morphologies of honokiol-treated *Cryptocaryon irritans* tomonts stained and unstained with Annexin V-FITC/PI. The *C. irritans* tomonts were treated with honokiol at 0.0, 0.6, 1.0, 2.0, 4.0, and 8.0 μg/ml for 8 h. The treated tomonts were incubated with Annexin V-FITC and a PI probe and observed under a fluorescence inversion microscope (DMi8 + DFC7000T, Leica Microsystems, Germany). The results show that all the honokiol-treated *C. irritans* tomonts’ cytoplasms obviously shrank and were stained with Annexin V-FITC. When the concentration of honokiol was > 4.0 μg/ml, the treated *C. irritans* tomonts’ cytoplasms were irregularly condensed, became hyaline, and were stained by PI. **a**–**f**: Morphologies of *C. irritans* tomonts respectively treated with 0.0, 0.6, 1.0, 2.0, 4.0, and 8.0 μg/ml honokiol for 8 h. **g**–**l**: Morphologies of *C. irritans* tomonts respectively treated with 0.0, 0.6, 1.0, 2.0, 4.0, and 8.0 μg/ml honokiol for 8 h and observed at Ex/Em = 488/525 nm. **m**–**r**: Morphologies of *C. irritans* tomonts respectively treated with 0.0, 0.6, 1.0, 2.0, 4.0, and 8.0 μg/ml honokiol for 8 h and observed at Ex/Em = 488/630 nm. **s**–**x**: Overlapping morphology photos of *C. irritans* tomonts respectively treated with 0.0, 0.6, 1.0, 2.0, 4.0, and 8.0 μg/ml honokiol for 8 h and recorded at Ex/Em = 488/525 nm and Ex/Em = 488/630 nm. All bars = 300 μm

**Fig. 2:**
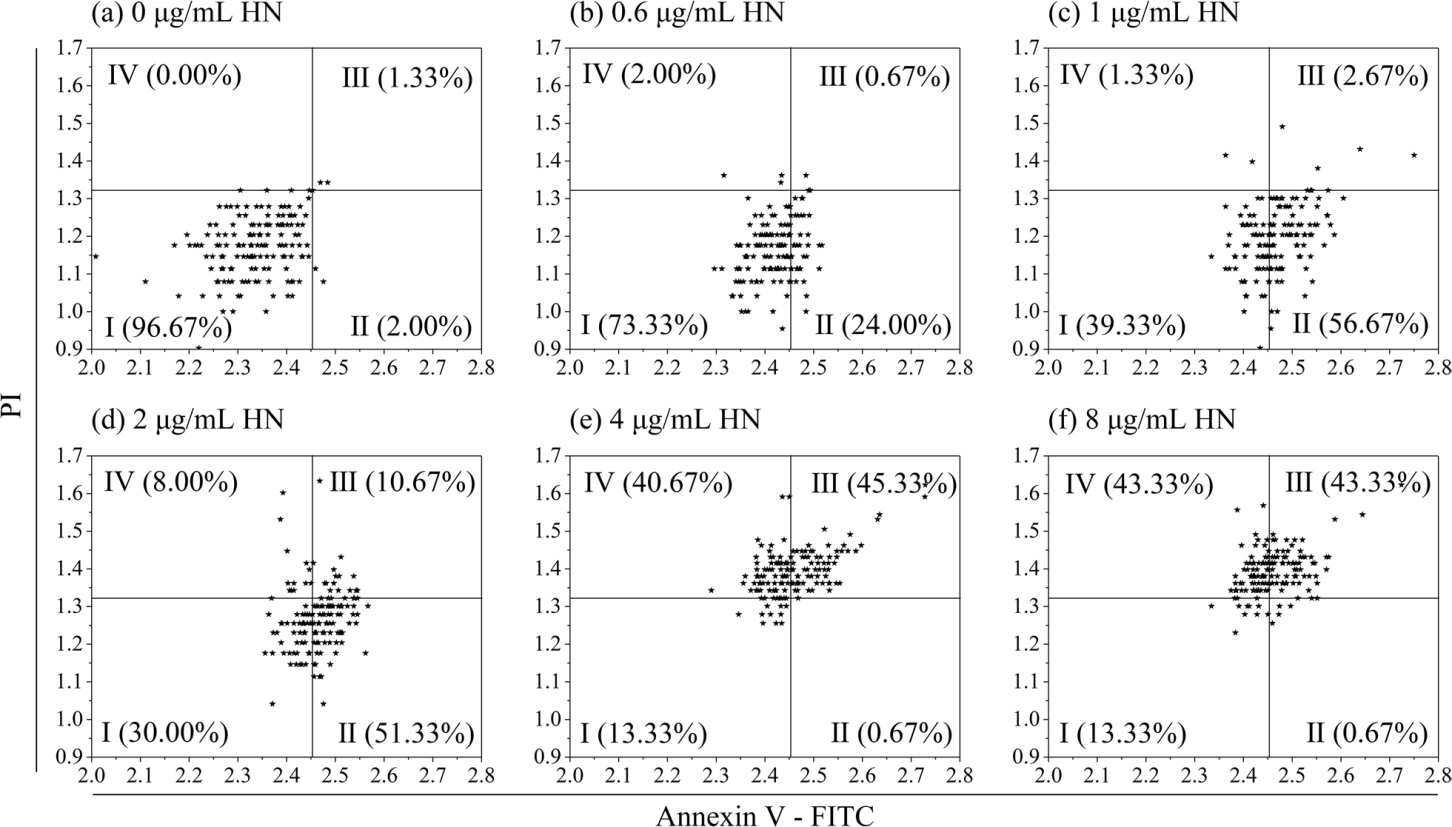
Four-quadrant apoptosis diagrams of *Cryptocaryon irritans* tomonts treated with honokiol at various concentrations for 8 h. The *C. irritans* tomonts were treated with honokiol at 0.0, 0.6, 1.0, 2.0, 4.0, and 8.0 μg/ml for 8 h. The treated tomonts were incubated with Annexin V-FITC and a PI probe and analyzed with a fluorescence microplate reader (BioTek Synergy H1, BioTek Instruments, Inc., USA). The results show that with the increase in honokiol concentration, the PADR began to increase at 0.6 μg/ml and reached the highest level at 1.0 μg/ml, while the AADR and NER began to increase at 2.0 μg/ml

The morphologies of the unstained and AnnexinV-FITC/PI-stained *C. irritans* tomonts treated with honokiol are given in Fig. 1, showing that all the honokiol-treated *C. irritans* tomonts' cytoplasms obviously shrank, their cell membranes were separated from cysts, and they were stained with Annexin V-FITC (showing green fluorescence) in a dose-dependent manner; this indicates phosphatidylserine valgus in cell membrane (a typical cell apoptosis characteristic). When the concentration of honokiol was > 4.0 μg/ml, the treated *C. irritans* tomonts' cytoplasms were irregularly condensed, became hyaline, and were stained by PI (showing red fluorescence), which indicates their cell membranes were damaged (a typical characteristic of middle- and late-stage cell apoptosis or necrosis). Four-quadrant apoptosis diagrams are given in Fig. 2, showing that with the increase in honokiol, the PADR began to increase at a concentration of 0.6 μg/ml. It reached its highest level when the honokiol concentration was 1.0 μg/ml and then decreased, while the AADR and NER began to increase at a concentration of 2.0 μg/ml.

### Effect of honokiol on [Ca^2+^]_i_ concentration, Δ***Ψ***m, ROS, QDF, and caspase-3/8/9 activities

**Fig. 3:**
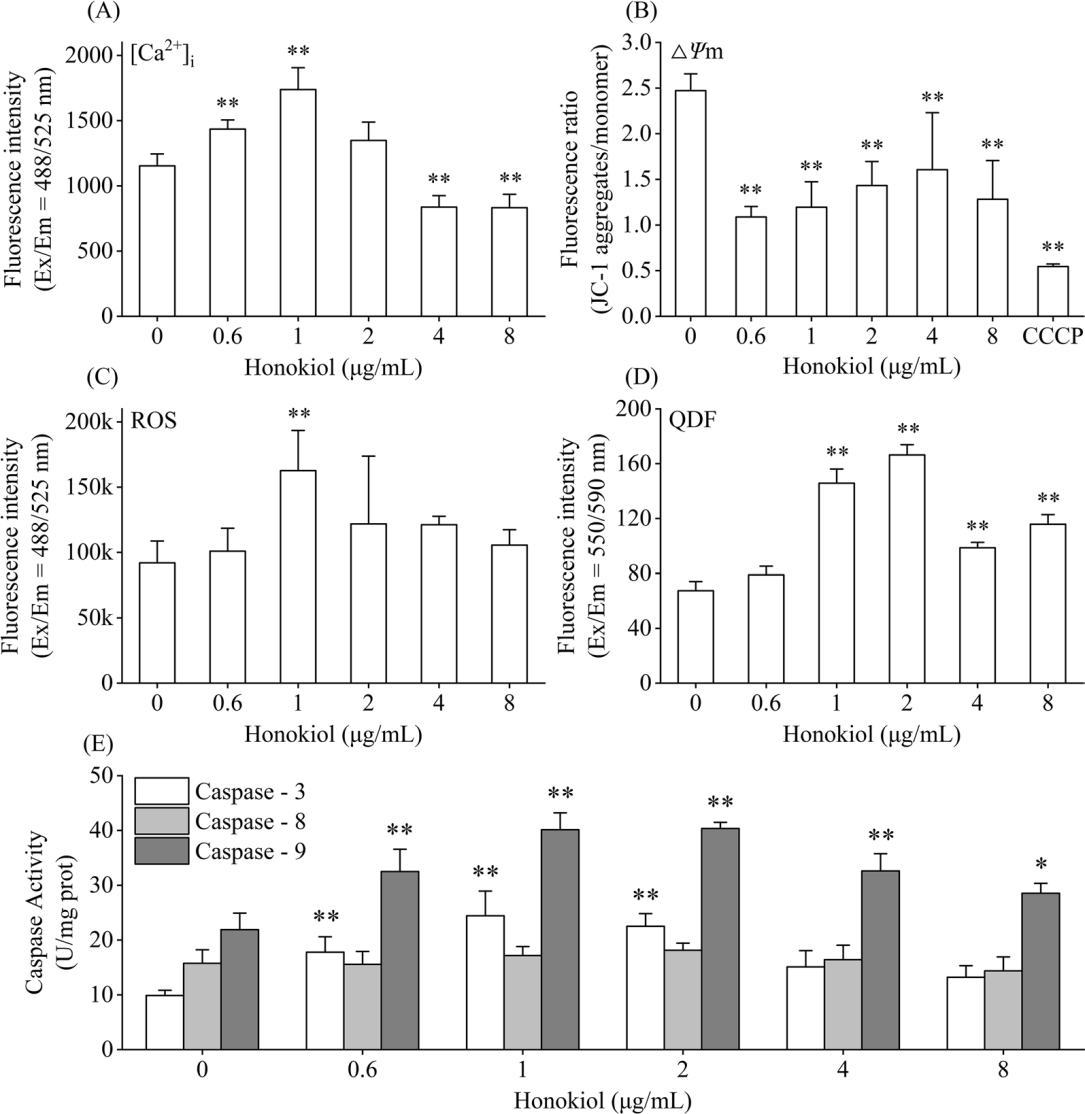
Effects of the 8 h treatment of honokiol at various concentrations on the [Ca^2+^]_i_ concentration, Δ*Ψ*m, ROS, QDF, and caspase-3/8/9 activities in *Cryptocaryon irritans* tomonts. The *C. irritans* tomonts were treated with honokiol at 0.0, 0.6, 1.0, 2.0, 4.0, and 8.0 μg/ml for 8 h, and the [Ca^2+^]_i_ concentration, Δ*Ψ*m, ROS, QDF, and caspase-3/8/9 activities were determined. The results show that the [Ca^2+^]_i_ concentration, Δ*Ψ*m, ROS, and the caspase-3/9 activities all reached the highest significant levels when the honokiol was 1.0 μg/ml, and the QDF reached the highest significant level when the honokiol was 2.0 μg/ml. **a** Effect of honokiol on [Ca^2+^]_i_ concentration in *C. irritans* tomonts. **b** Effect of honokiol on Δ*Ψ*m in *C. irritans* tomonts. **c** Effect of honokiol on ROS in *C. irritans* tomonts. **d** Effect of honokiol on QDF in *C. irritans* tomonts. **e** Effect of honokiol on caspase-3/8/9 activities in *C. irritans* tomonts. The results are expressed as mean ± SD, *n* = 5. *Significant difference from the control sample (0.0 μg/ml), *P* < 0.05. **Highly significant difference from the control sample (0.0 μg/ml), *P* < 0.01

The [Ca^2+^]_i_ concentration and the Δ*Ψ*m, ROS, QDF, and caspase-3/8/9 activities in the *C. irritans* tomonts treated with honokiol at various concentrations are given in Fig. 3. As shown in Fig. 3A, with the increase in the honokiol concentration, the [Ca^2+^]_i_ concentration increased to a level significantly higher than that of the control sample at 0.6 μg/ml. It reached the highest level at 1 μg/ml, returned to the level of the control sample at 2.0 μg/ml, and then decreased to a level significantly lower than that of the control sample when the honokiol concentration increased > 4.0 μg/ml. As shown in Fig. 3B, the Δ*Ψ*m decreased to a level significantly lower than that of the control sample when the honokiol concentration was > 0.6 μg/ml. As shown in Fig. 3C, with the increase of the honokiol concentration, the ROS increased to a level significantly higher than that of the control sample at 1.0 μg/ml and then returned to the level of the control sample. As Fig. 3D shows, with the increase of the honokiol concentration, the QDF began to increase at 0.6 μg/ml, increased to a level significantly higher than that of the control sample at 1.0 μg/ml, reached the highest level at 2.0 μg/ml, and then decreased, but the level remained significantly higher than that of the control sample when the honokiol concentration increased above 4.0 μg/ml. As shown in Fig. 3E, with the increase of the honokiol concentration, both the caspase-3/9 activities began to increase to levels significantly higher than those of the control sample at 0.6 μg/ml and reached the highest levels at 1.0 μg/ml. The activity of caspase-3 gradually returned to the level of the control sample when the honokiol concentration was ≥ 4.0 μg/ml, while the activity of caspase-9 remained at a level higher than that of the control sample, and the activity of caspase-8 always remained at the level of the control sample.

### Effects of honokiol treatment time on *C. irritans* tomont apoptosis-like death

**Fig. 4:**
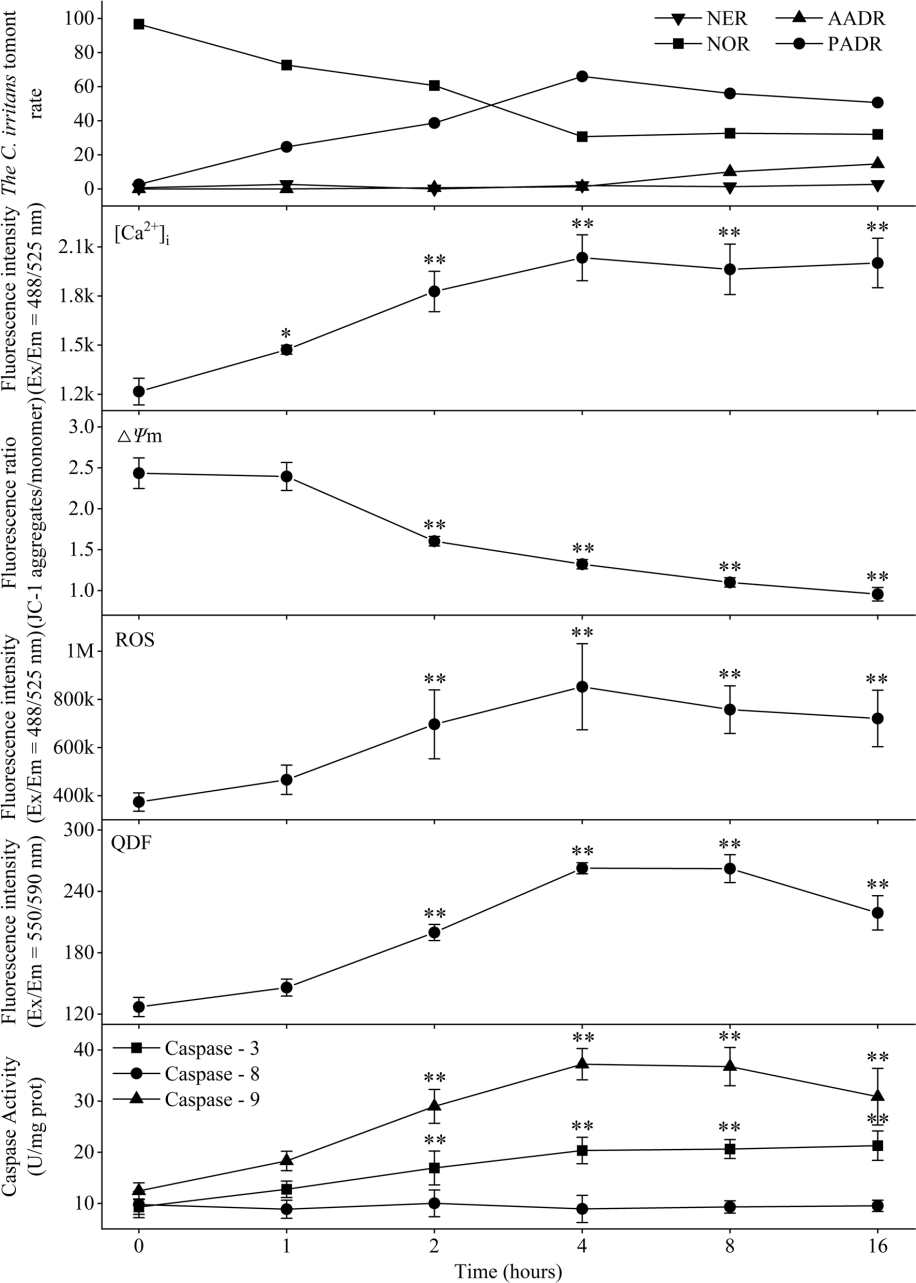
Effect of honokiol treatment time on *Cryptocaryon irritans* tomont apoptosis-like death. The *C. irritans* tomont treated with 1 μg/ml honokiol at 0, 1, 2, 4, 8, and 16 h and their NOR, PADR, AADR, NER, [Ca^2+^]_i_ concentration, Δ*Ψ*m, ROS, QDF, and caspase-3/8/9 activities were determined. The result shows that the [Ca^2+^]_i_ concentration began to increase significantly at 1 h, and then the ROS, QDF, and caspase-3/9 activities began to increase significantly and the Δ*Ψ*m began to decrease significantly at 2 h; the highest PADR was obtained at 4 h. The results are expressed as mean ± SD, *n* = 5. *Significant difference from the control sample (0.0 μg/ml), *P* < 0.05. **Highly significant difference from the control sample (0.0 μg/ml), *P* < 0.01

The results found in “Effect of honokiol on [Ca^2+^]_i_ concentration, Δ*Ψ*m, ROS, QDF, and caspase-3/8/9 activities” showed that *C. irritans* tomonts had the highest PADR, [Ca^2+^]_i_ concentration, ROS, caspase-3/9 activities, and higher QDF, and the lowest NER when the honokiol concentration was 1.0 μg/ml. Therefore, 1.0 μg/ml was considered the optimum honokiol concentration for inducing *C. irritans* tomont apoptosis-like death. Together with the extension of the treatment time, the changes in the NOR, PADR, AADR, NER, [Ca^2+^]_i_ concentration, Δ*Ψ*m, ROS, QDF, and the caspase-3/8/9 activities for the *C. irritans* tomonts are given in Fig. 4, showing that, with the extension of the treatment time, the [Ca^2+^]_i_ concentration and PADR began to increase significantly at 1 h, followed by ROS and QDF. The caspase-3/9 activities began to increase significantly, and the Δ*Ψ*m began to decrease significantly at 2 h. The [Ca^2+^]_i_ concentration, PADR, ROS, QDF, and caspase-3/9 activities reached the highest levels at 4 h and then slightly decreased but remained at levels significantly higher than those at 0 h, while the Δ*Ψ*m continued to decrease at 16 h, and the AADR began to significantly increase at 8 h. The NER and the activity of caspase-8 showed nonsignificant changes at 16 h. These results suggest that the optimum treatment time for 1.0 μg/ml honokiol inducing *C. irritans* tomonts apoptosis-like death is 4 h. The inhibition rate of tomont hatching was 52.38% after treatment with 1.0 μg/ml honokiol for 4 h.

### Effects of honokiol on the mRNA expression of apoptosis-related genes

**Fig. 5:**
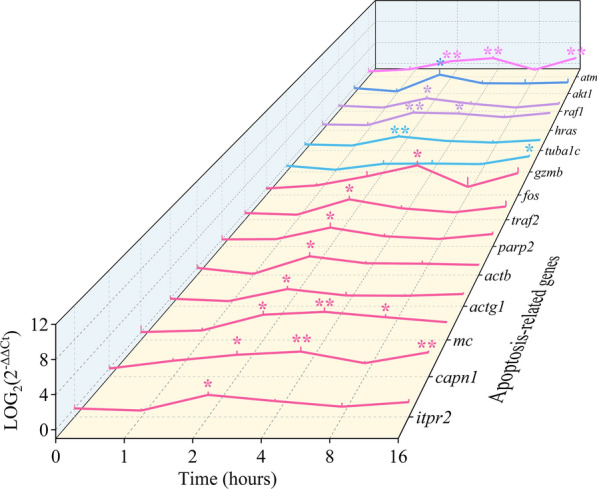
mRNA expression of apoptosis-related genes in *Cryptocaryon irritans* tomonts treated with honokiol. The mRNA expression of the apoptosis-related genes in *C. irritans* tomonts were respectively treated with 1.0 μg/ml honokiol at 0, 1, 2, 4, 8, and 16 h. The results show that a total of 14 genes increased significantly at different times. The results are expressed as mean ± SD, *n* = 3. *Significant difference from the control (0.0 μg/ml), *P* < 0.05. **Highly significant difference from the control (0.0 μg/ml), *P* < 0.01

Among the 61 investigated apoptosis-related genes, 14 were significantly upregulated (shown in Fig. 5). The fluorescence quantitative melting curves of the 14 genes are shown in Additional file 2: Fig. S1. Among the 14 upregulated genes, *itpr2*, *capn1*, *mc*, *actg1*, *actb*, *parp2*, *traf2*, and *fos* were enriched in the pathway related to apoptosis induced by the disruption of the [Ca^2+^] homeostasis in ER. Among the eight genes, *fos* was significantly upregulated at 4 h, while the other genes were significantly upregulated within 2 h. Gene *gzmb*, enriched in the Granzyme B pathway, was significantly upregulated at 16 h, and gene *tuba1c*, enriched downstream of the Granzyme B pathway, was significantly upregulated at 2 h. Genes *hras* and *raf1*, enriched in the MAPK signaling pathway, were significantly upregulated at 2 h, and gene *hras was* also significantly upregulated at 4 h. Gene *akt1*, enriched in the PI3K-Akt signaling pathway, was significantly upregulated at 2 h. Gene *atm*, the upstream regulatory of the p53 signaling pathway, was significantly upregulated at 2, 4, and 16 h.

## Discussion

The apoptosis-like death pathway has been found in many protozoa, such as Leishmania, *P. falciparum*, *T. thermophila*, *T. cruzi*, *B. hominis*, *T. gondii,* and *I. multifiliis* [12–21]. *Ichthyophthirius multifiliis* is the pathogen of freshwater white spot disease, the morphology and life cycle of which are similar to those of *C. irritans* [1]. It has been reported that fish skin antibodies could cause *I. multifiliis* apoptosis-like phenomena, such as PS externalization and chromatin condensation [16]. It has also been reported that malachite green could cause *I. multifiliis* apoptosis-like phenomena, such as mitochondrial swelling, mitochondrial membrane integrity destruction, ribosome number change, and PS externalization through the PI3K-Akt signal pathway [21]. In this study, honokiol was demonstrated to cause significant *C. irritans* tomont cytoplasm atrophy, cell volume reduction, PS externalization, a significant increase in QDF, [Ca^2+^]_i_ concentration, ROS and caspase-3/9 activities, a significant decrease in Δ*Ψ*m, and significant upregulation of the mRNA expression of the 14 apoptosis-related genes; this strongly suggests that *C. irritans* tomonts have a form of regulated apoptosis-like death. The apoptosis-like death named by the Nomenclature Committee on Cell Death (NCCD) resembles the apoptosis of metazoans [35] and has been considered an ideal strategy to prevent and treat parasitic diseases [11, 36]. Although this study has proved that apoptosis-like death exists in *C. irritans* tomonts, which provides a potential new way to treat marine fish white spot disease with the advantages of a lower probability of drug resistance and adverse effects, further studies are needed to uncover and confirm the mechanism of honokiol inducing *C. irritans* tomont apoptosis-like death.

**Fig. 6:**
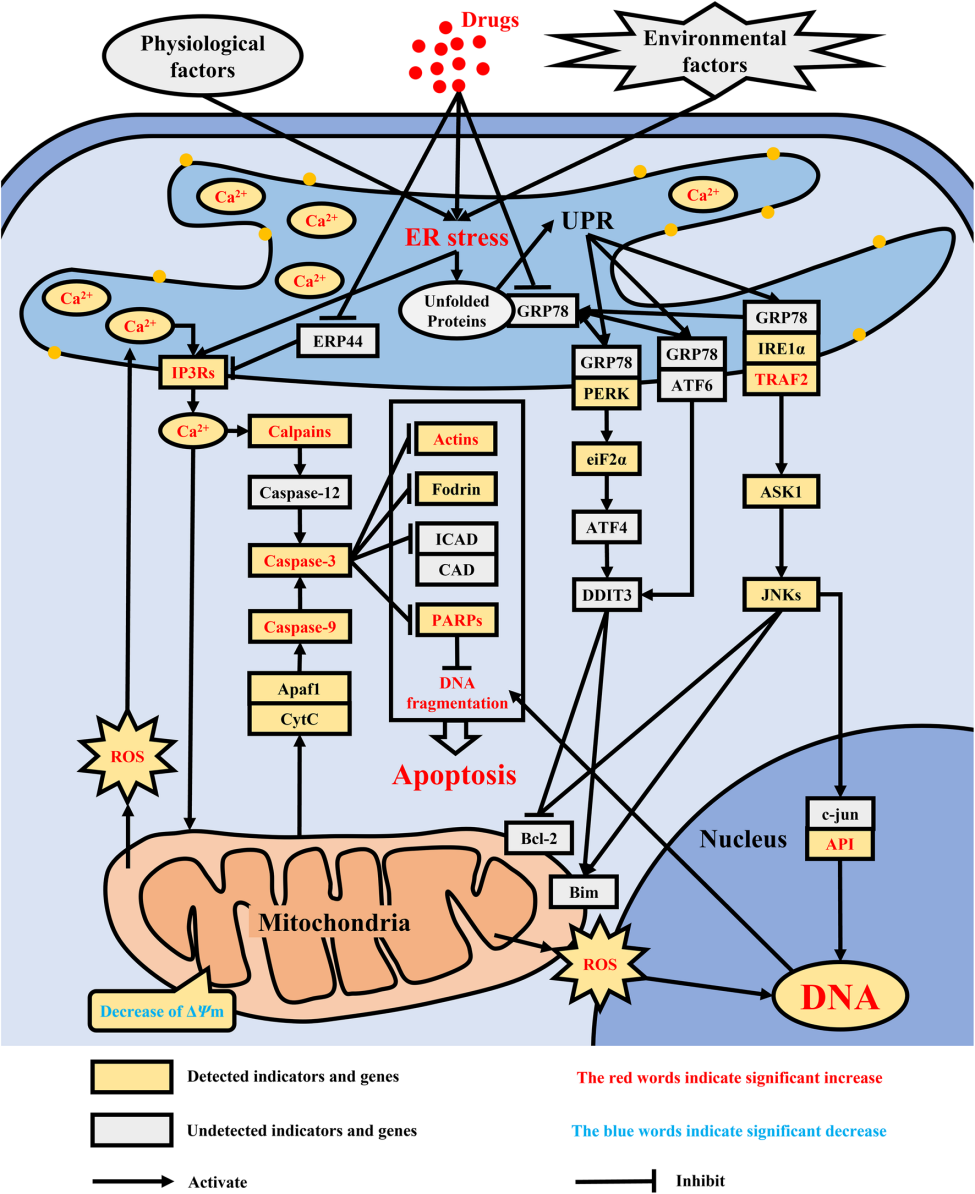
Regulatory mechanism of apoptosis induced by ER stress. This figure shows that the classic ER stress pathway induces cell apoptosis. Apaf1, Cytc, Fodrin, PERK, eiF2α, IRE1α, TRAF2, ASK1, and API were coded with *apaf1*, *cycs*, *sptan1*, *eif2ak3*, *eif2s1*, *ern1*, *traf2*, *map3k5*, and *fos*, respectively. Beta-actin and γ-actin were respectively coded with *actb* and *actg1*. Calpains included the calpain-1 catalytic subunit and the calpain-2 catalytic subunit, which were coded with *capn1* and *capn2*. PARPs include poly [ADP-ribose] polymerase 2 and poly [ADP-ribose] polymerase 4, which were coded with *parp2* and *parp4*. JNKs include mitogen-activated protein kinase 9 and mitogen-activated protein kinase 10, which were respectively coded with *mapk9* and *mapk10*. IP3Rs include inositol 1,4,5-trisphosphate receptor type 2 and inositol 1,4,5-trisphosphate receptor type 3, which were respectively coded with *itpr1*, *itpr2*, and *itpr3*. The mRNA expression of *itpr2*, *capn1*, *mc*, *actg1*, *actb*, *parp2*, *traf2*, and *fos* increased significantly in this study

Honokiol, as one of the main active components of *M. officinalis*, has been reported to induce apoptosis of several cells, such as neuroblastoma cells, A549 cells, 95-D cells, and human chondrosarcoma cells, fungi such as *C. albicans*, and protozoan parasites such as Leishmania via the ER stress pathway [8, 27–30, 36–39]. The reported ER stress pathway inducing cell apoptosis is summarized in Fig. 6, which shows that drugs such as honokiol and physiological or environmental factors can cause excessive or aberrant ER stress [30, 40–42]. Excessive or aberrant ER stress leads to Ca^2+^ release from ER via the inositol 1,4,5-trisphosphate receptor (IP3Rs) and to unfolded protein response (UPR) accumulation [43–47]. Cho et al. [48] reported that honokiol could promote [Ca^2+^]_i_ release and increase [Ca^2+^]_i_ concentration by inhibiting the activity of endoplasmic reticulum protein 44 (ERP44), which has inhibitory activity on the calcium channel IP3Rs [49]. Similarly, the [Ca^2+^]_i_ concentration first significantly increased in *C. irritans* tomonts in this study. The increase of Ca^2+^ can cause two significant reactions, Reaction I and Reaction II. In Reaction I, the increase of Ca^2+^ can provoke the cascade reaction of caspases, including calpains, caspase-12, and caspase-3 [50–52]. Coinciding with Reaction I, the mRNA expression of gene *capn1* coding calpain was significantly upregulated and the activity of caspase-3 significantly increased in *C. irritans* tomonts. Via blasting in the *C. irritans* genome, gene *caspase-12* coding caspase-12 was not found, but gene *mc* coding metacaspase was found, and its mRNA expression was also significantly upregulated. Metacaspase has been reported to have an apoptosis regulation function in protozoa such as Leishmania and Plasmodium, which is similar to caspase-12 in metazoa [11, 53, 54]. In Reaction II, the increase of [Ca^2+^]_i_ can also decrease the Δ*Ψ*m of mitochondria and then promote ROS production and release from mitochondria [44, 55, 56]. An increase in ROS can further promote [Ca^2+^]_I_ release and increase QDF, and then the mitochondria sequentially activate caspase-9 and caspase-3 via apoptotic protease-activating factor 1 (Apaf1) combined with cytochrome c (CytC) [45, 57]. In agreement with Reaction II, honokiol has been proven to significantly decrease Δ*Ψ*m of mitochondria and increase ROS production, the activities of caspase-9 and caspase-3, and QDF in *C. irritans* tomonts. The activated caspase-3 can hydrolyze and deactivate actins and poly ADP-ribose polymerase (PARPs) [58–60]. Actins are involved in the maintenance of the cytoskeleton. Therefore, hydrolyzed actins cause cytoplasm atrophy and cell volume reduction [61–63], while hydrolyzed PARPs cause irreversible DNA damage and QDF increase [64–66]. In supporting these phenomena, this article has proven that honokiol could significantly upregulate the mRNA expressions of genes *actg1*, *actb*, and *parp2*, which respectively code actins and PARPs, cause *C. irritans* tomont cytoplasm atrophy and cell volume reduction, and increase the QDF [67–69]. Besides this apoptosis pathway, caused by ER stress via Ca^2+^, the ER stress also leads to the accumulation of UPR. Under homeostatic conditions, proteins involved in the UPR, protein kinase RNA-like endoplasmic reticulum kinase (PERK), activating transcription factor 6 (ATF6), and inositol-requiring enzyme 1α (IRE1α) are bound to glucose-regulated protein 78 (GRP78) by their ER lumen domains, keeping them inactive. With the accumulation of UPR, causing GRP78 to leave PERK, ATF6, and IRE1α, the activation of these proteins is induced [45–47] (Fig. 6). The activated PERK activates the cascade reaction, including eukaryotic translation initiation factor 2 subunit alpha (eiF2α), activating transcription factor 4 (ATF4), and DNA damage inducible transcript 3 (DDIT3). DDIT3 can decrease the expression of anti-apoptosis proteins such as B-cell lymphoma 2 (Bcl-2) and increase the expression of pro-apoptosis proteins such as Bcl-2 interacting mediator of cell death (Bim). Then, the mitochondria sequentially activate caspase-9 and caspase-3 via Apaf1 combined with CytC. Finally, cell apoptosis is induced [46]. The activated ATF6 also activates DDIT3 and induces apoptosis via the mitochondria pathway. The activated IRE1α, combined with TNF receptor-associated factor 2 (TRAF2), activates cascade reactions, including apoptosis signal-regulating kinase 1 (ASK1) and c-jun n-terminal kinase (JNKs). JNKs are responsible for phosphorylation of the mitochondrial proteins Bim (pro-apoptosis) and Bcl2 (anti-apoptosis), which are activated and inhibited, respectively. Then, the activated JNKs induce the mitochondria apoptosis pathway or upregulate the expression of the jun proto-oncogene (c-jun) and fos proto-oncogene (API), increase the QDF, and induce cell apoptosis [45–47]. Although the mRNA expressions of *traf2* and *fos* in this pathway were significantly upregulated by honokiol, those of *eif2ak3*, *eif2s1*, *ern1*, *map3k5*, *mapk9*, and *mapk10* showed nonsignificant changes when *C. irritans* tomonts were treated with honokiol. This suggests that honokiol might not induce *C. irritans* tomont apoptosis via this pathway. In summary, the results of this study suggest that honokiol might inhibit the activity of ERP44 and unlimited IP3R release [Ca^2+^]_i_, disrupt [Ca^2+^]_i_ homeostasis in ER, and then induce *C. irritans* tomont apoptosis-like death by caspase cascade or mitochondrial pathway. However, further studies such as the transcriptomic or proteomic analyses are needed to confirm this suggestion.

## Conclusion

This article showed that honokiol can induce *C. irritans* tomont apoptosis-like death and suggested that honokiol may disrupt [Ca^2+^]_i_ homeostasis in ER and then induce *C. irritans* tomont apoptosis-like death by caspase cascade and mitochondrial pathway. This might represent a novel therapeutic intervention for *C. irritans* infection. Next, to further research on safe and efficient anti-*C. irritans* drugs, the intracellular target of honokiol in *C. irritans* needs to be verified.

### Supplementary Information

**Additional file 1: Table S1. **The 61 apoptosis-related genes and their primers used in this study.

**Additional file 2: Fig. S1. **The fluorescence quantitative melting curves of the 14 significantly differentially expressed genes.

## Data Availability

The data supporting the conclusions of this article are included within the article.

## Abbreviations

*C. irritans*: *Cryptocaryon irritans*
ER: Endoplasmic reticulum
NOR: Normal rate
PADR: Prophase apoptosis-like death rate
AADR: Anaphase apoptosis-like death rate
NER: Necrosis rate
[Ca^2+^]_i_ concentration: Intracellular calcium concentration
Δ*Ψ*m: Mitochondrial membrane potential
ROS: Reactive oxygen species
QDF: Quantity of DNA fragmentations
IP3Rs: Inositol 1,4,5-trisphosphate receptor
UPR: Unfolded protein response
ERP44: Endoplasmic reticulum protein 44
Apaf1: Apoptotic protease-activating factor 1
CytC: Cytochrome c
PARPs: Poly ADP-ribose polymerase
PERK: Protein kinase RNA-like endoplasmic reticulum kinase
ATF6: Activating transcription factor 6
IRE1α: Inositol-requiring enzyme 1α
GRP78: Glucose-regulated protein 78
eiF2α: Eukaryotic translation initiation factor 2 subunit alpha
ATF4: Activating transcription factor 4
DDIT3: DNA damage inducible transcript 3
Bcl-2: B-cell lymphoma 2
Bim: Bcl-2 interacting mediator of cell death
TRAF2: TNF receptor-associated factor 2
ASK1: Apoptosis signal-regulating kinase 1
JNKs: C-jun n-terminal kinase
c-jun: Jun proto-oncogene
API: Fos proto-oncogene
