# Supplementary material for: Honokiol induces apoptosis-like death in Cryptocaryon irritans Tomont
Source: Parasit Vectors. 2023 Aug 16;16:287. doi: 10.1186/s13071-023-05910-1 (PMC10428556; doi:10.1186/s13071-023-05910-1)
Supplement: Supplementary file 1 — Additional file 1: Table S1. The 61 apoptosis-related genes and their primers used in this study. [file 13071_2023_5910_MOESM1_ESM.docx]

**Table S1.** **The 61 apoptosis-related genes and their primers used in this study.**

| Number | Gene ID | Gene Symbol | Gene Description | Primer Sequence (5’-3’) | |
| --- | --- | --- | --- | --- | --- |
| 1 | evm.model.contig_130.3 | *itpr2* | Inositol 1,4,5-trisphosphate receptor type 2 | Forward | CCACCCCTCATGAAAACTGGA |
|  |  |  |  | Reverse | CACGATGTTTCCCACCTTTGA |
| 2 | evm.model.contig_2127.4 | *capn1* | Calpain-1 catalytic subunit | Forward | GAGCCTGGAGTGACAGtgtg |
|  |  |  |  | Reverse | TCAGAGCTGACAAACGAGCA |
| 3 | evm.model.contig_184.1 | *mc* | Metacaspase | Forward | ATgtggATAGGTCTTGGCGC |
|  |  |  |  | Reverse | CCACAGCCACACTTGCATTC |
| 4 | evm.model.contig_943.3 | *actg1* | Actin, cytoplasmic 2 | Forward | TGAAGTCGGTTAGTCATTCGCA |
|  |  |  |  | Reverse | TCGTTTTGGGAGGATTCAGCA |
| 5 | evm.model.contig_1609.1 | *actb* | Actin, cytoplasmic 1 | Forward | ACCCATTCCACCAGAAGACC |
|  |  |  |  | Reverse | AGTCCTTGTTCTCCCTTGACT |
| 6 | evm.model.contig_366.8 | *parp2* | Poly [ADP-ribose] polymerase 2 | Forward | TGATCGTGATGGGGAAGCTG |
|  |  |  |  | Reverse | AGGAGGAGCAATTTTCAACCCA |
| 7 | evm.model.contig_524.5 | *traf2* | TNF receptor-associated factor 2 | Forward | TCAGAGTTCCCATTGATTTGTCCT |
|  |  |  |  | Reverse | CTTTGCAGATTTCGCAGAGCA |
| 8 | evm.model.contig_1311.1 | *fos* | Protein c-Fos | Forward | CACCAGTGCCTCTACATCCC |
|  |  |  |  | Reverse | TGCAGGGAGTGATCCGATTG |
| 9 | evm.model.contig_2050.1 | *gzmb* | Granzyme B | Forward | TCCTTTCAGGGTGACTCAGGA |
|  |  |  |  | Reverse | GACACCAGGGTAGTAAGCGT |
| 10 | evm.model.contig_564.3 | *tuba1c* | Tubulin alpha-1C chain | Forward | AAGGGGAAGTCGATGCAACA |
|  |  |  |  | Reverse | GCTACTTGTATTGAAGCTGGTCC |
| 11 | evm.model.contig_91.7 | *hras* | GTPase Hras | Forward | TGGCTTGCAGAAGTAGAGAAA |
|  |  |  |  | Reverse | GCCAATTCCTGACCTTCTTCAAAT |
| 12 | evm.model.contig_745.4 | *raf1* | RAF proto-oncogene serine/threonine-protein kinase | Forward | GCAACAGGAGAGATCCCATACC |
|  |  |  |  | Reverse | GGTGTCCAGTTTTCGGAATCG |
| 13 | evm.model.contig_708.3 | *akt1* | RAC-alpha serine/threonine-protein kinase | Forward | ACTGAGGAGACACAAGACGT |
|  |  |  |  | Reverse | AGCCAATCCGAAATCCGTGA |
| 14 | evm.model.contig_940.1 | *atm* | Serine-protein kinase ATM | Forward | TTTCCCCGAAACTCCCTGTC |
|  |  |  |  | Reverse | ACTCGTTCATCTTGGCGGAG |
| 15 | evm.model.contig_29.13 | *akt3* | RAC-gamma serine/threonine-protein kinase | Forward | AGGGGATCATTTGGAAAGGTGA |
|  |  |  |  | Reverse | ACTAAAAACGGATGGTCAGCT |
| 16 | evm.model.contig_141.9 | *apaf1* | Apoptotic protease-activating factor 1 | Forward | CTTACTCCCGATCCGTGCAT |
|  |  |  |  | Reverse | TTGTTTCCTGTGGTGGCAAA |
| 17 | evm.model.contig_1801.1 | *birc2* | Baculoviral IAP repeat-containing protein 2 | Forward | AGTGTATTTGCTAATTGTGGGC |
|  |  |  |  | Reverse | AAAGGACATTAAATTGGCTGAGGC |
| 18 | evm.model.contig_1371.4 | *birc3* | Baculoviral IAP repeat-containing protein 3 | Forward | CCTCCGATAAACATCAAGAAGACC |
|  |  |  |  | Reverse | GGAGTAAGAGCATCAAAGTGAAAAG |
| 19 | evm.model.contig_196.9 | *capn2* | Calpain-2 catalytic subunit | Forward | AGGCTCTTCCTTACAAGCATCA |
|  |  |  |  | Reverse | CCCCTGCATGAGCTACACTTT |
| 20 | evm.model.contig_852.1 | *chuk* | Inhibitor of nuclear factor kappa-B kinase subunit alpha | Forward | GGACACTTTTTCAAAACAGGCG |
|  |  |  |  | Reverse | TGCCTACCTTTCTGTCTCCT |
| 21 | evm.model.contig_871.3 | *ctsb* | Cathepsin B | Forward | GATCATGCTGGGCTTTCGGA |
|  |  |  |  | Reverse | CCATCTCCACAGGAAGTACAACA |
| 22 | evm.model.contig_141.4 | *ctsc* | Dipeptidyl peptidase 1 | Forward | ACCCAGAAGTCAAAGCCTGG |
|  |  |  |  | Reverse | TCTCCCCAATCTTCTCCCCA |
| 23 | evm.model.contig_923.4 | *ctsd* | Cathepsin D | Forward | AGTAGGCAAGGGAGTTGGAGA |
|  |  |  |  | Reverse | CACCGAGGAGTTGTGCATGA |
| 24 | evm.model.contig_95.5 | *ctsf* | Cathepsin F | Forward | CTGGGCATTTTCAGCAGTCG |
|  |  |  |  | Reverse | AGGCGTCGGTCATTAAACCT |
| 25 | evm.model.contig_291.4 | *ctsl* | Procathepsin L | Forward | TCATGCTGGGCTTTTTCCAC |
|  |  |  |  | Reverse | CCTCACATCCGTAATTGCCG |
| 26 | evm.model.contig_2299.1 | *cycs* | Cytochrome c | Forward | CCAGCAGCAGGAAGAGAAGT |
|  |  |  |  | Reverse | TCCCATGACTCCTCCCAAAC |
| 27 | evm.model.contig_30.13 | *eif2ak3* | Eukaryotic translation initiation factor 2-alpha kinase 3 | Forward | AGGAGAAGGAGGATTTGGAAAAGT |
|  |  |  |  | Reverse | TCCACTTAACATGGCAACTTCT |
| 28 | evm.model.contig_1054.7 | *eif2s1* | Eukaryotic translation initiation factor 2 subunit 1 | Forward | AATGAAAGTCGCCCCCAGAG |
|  |  |  |  | Reverse | CCACCCCAATATCAGCATCCA |
| 29 | evm.model.contig_682.6 | *ern1* | Serine/threonine-protein kinase/endoribonuclease IRE1 | Forward | TGTCGGCTCTCTCAACTCTAT |
|  |  |  |  | Reverse | GGGCTTGAAACGGAATAGTACC |
| 30 | evm.model.contig_177.2 | *gadd45g* | Growth arrest and DNA damage-inducible protein  GADD45 gamma | Forward | CAGAAAATTGCGATGAATCCCACT |
|  |  |  |  | Reverse | GCAAATTCCAATCCATTCACCCA |
| 31 | evm.model.contig_1.59 | *htra2* | Serine protease HTRA2, mitochondrial | Forward | GTGGGGGATTGGGTTTTTGC |
|  |  |  |  | Reverse | AGCCGCATCTGTTTGGAAGT |
| 32 | evm.model.contig_1078.3 | *ikbkb* | Inhibitor of nuclear factor kappa-B kinase subunit beta | Forward | TGTCCATAGAGATTTAAAGCCAGCT |
|  |  |  |  | Reverse | TTCCATTCCTTGATCCACCACT |
| 33 | evm.model.contig_1545.1 | *itpr1* | Inositol 1,4,5-trisphosphate receptor type 1 | Forward | AAGGTGGGGTGAATTTGTAGT |
|  |  |  |  | Reverse | TCACTGTCTGGACCTTGGGT |
| 34 | evm.model.contig_64.6 | *itpr3* | Inositol 1,4,5-trisphosphate receptor type 3 | Forward | TCTAATGAGGGAAGCACGACT |
|  |  |  |  | Reverse | ACGGTAGCACAATCGGAAGT |
| 35 | evm.model.contig_425.1 | *kras* | GTPase Kras | Forward | ACCCTACATTACAAGACACATTTAGA |
|  |  |  |  | Reverse | TCCATTCCCTTCTTTTATCCAATGA |
| 36 | evm.model.contig_523.2 | *lmna* | Prelamin-A/C | Forward | TGAGAGATCCGTTTTGCCTTCT |
|  |  |  |  | Reverse | TCCTTTCCCTTGTCCACTCTC |
| 37 | evm.model.contig_757.10 | *lmnb1* | Lamin-B1 | Forward | GGAGGGACAATGCATGACCA |
|  |  |  |  | Reverse | GACTGCAGCCTGGACTCTTT |
| 38 | evm.model.contig_300.2 | *map2k1* | Dual specificity mitogen-activated protein kinase kinase 1 | Forward | AGTTAATTCGGATGGAGAGGTCA |
|  |  |  |  | Reverse | AATCATTGTTCCTGCCCAAGT |
| 39 | evm.model.contig_490.3 | *map2k2* | Dual specificity mitogen-activated protein kinase kinase 2 | Forward | ACTCCATATTACATGGCACCAGA |
|  |  |  |  | Reverse | GCAAGCATCCAATAAAGTCAAACC |
| 40 | evm.model.contig_1510.2 | *map3k14* | Mitogen-activated protein kinase kinase kinase 14 | Forward | TCGGATTTGCTAAATTTATGGGGA |
|  |  |  |  | Reverse | TGAGCTAAGGACCAAATATCACA |
| 41 | evm.model.contig_7.21 | *map3k5* | Mitogen-activated protein kinase kinase kinase 5 | Forward | GGAGGGTTTAGAATATTTGCACTGG |
|  |  |  |  | Reverse | GGAGCCATCCAATTAGCAGT |
| 42 | evm.model.contig_165.7 | *mapk1* | Mitogen-activated protein kinase 1 | Forward | ACTGCTCCTGTTCTCACTGA |
|  |  |  |  | Reverse | ACAGACCACATATCAACAGCCT |
| 43 | evm.model.contig_1689.6 | *mapk10* | Mitogen-activated protein kinase 10 | Forward | GTCTCGGCTTGTATCGGCTT |
|  |  |  |  | Reverse | GGACAAACGGGGAAAGGACT |
| 44 | evm.model.contig_703.2 | *mapk3* | Mitogen-activated protein kinase 3 | Forward | CGAGAAACATAGAAGATGAGGATGAC |
|  |  |  |  | Reverse | GCAGCCAACACTCCAAATATCAA |
| 45 | evm.model.contig_187.1 | *mapk9* | Mitogen-activated protein kinase 9 | Forward | GTCTAATTTTGGGCGAGGAATCAA |
|  |  |  |  | Reverse | TTCTAGGCAGCTACTCCCGA |
| 46 | evm.model.contig_41.10 | *nfkb1* | Nuclear factor NF-kappa-B p105 subunit | Forward | AGCAACACCACTTCATTATGC |
|  |  |  |  | Reverse | GTGCAAAGGGGTATTTCCAAAA |
| 47 | evm.model.contig_2113.2 | *nfkbia* | NF-kappa-B inhibitor alpha | Forward | TCCTCTTCATTGGGCTTGCT |
|  |  |  |  | Reverse | TGCAAAGGTGTTAAGCCAGC |
| 48 | evm.model.contig_1665.2 | *nras* | GTPase Nras | Forward | TCCAATGGTTCTGGTAGCAAA |
|  |  |  |  | Reverse | TCCCGTTTTGGCACTTACTTCT |
| 49 | evm.model.contig_1364.5 | *parp4* | Protein mono-ADP-ribosyltransferase PARP4 | Forward | TCAGCATAGCCGCCTTTTCA |
|  |  |  |  | Reverse | CTTCTCTGCCCAGGTGTGTT |
| 50 | evm.model.contig_843.7 | *pdpk1* | 3-phosphoinositide-dependent protein kinase 1 | Forward | TTTCCGAGAGTGTTGCTGCT |
|  |  |  |  | Reverse | ACTCCATCCAAAGTCTGAAAGCT |
| 51 | evm.model.contig_1570.9 | *pidd1* | p53-induced death domain-containing protein 1 | Forward | TGTCCGAGACTGCATGTGAG |
|  |  |  |  | Reverse | GGTTTGGACTCCCCTCTTCG |
| 52 | evm.model.contig_2018.1 | *pik3ca* | Phosphatidylinositol 4,5-bisphosphate 3-kinase  catalytic subunit alpha isoform | Forward | TGGGCAGATCCTGAATAGGT |
|  |  |  |  | Reverse | TCTCACTCCCTCATCAGCAA |
| 53 | evm.model.contig_801.2 | *pik3cb* | Phosphatidylinositol 4,5-bisphosphate 3-kinase  catalytic subunit beta isoform | Forward | AGAAGCAAGTCCAGAAAGTCGA |
|  |  |  |  | Reverse | GCTTTGCACACAGGATTAGGTC |
| 54 | evm.model.contig_199.1 | *prf1* | Perforin-1 | Forward | ATAGTGAAATGGGGGCAGGG |
|  |  |  |  | Reverse | CTCTTTGGCATTGGAGGAGGT |
| 55 | evm.model.contig_1516.5 | *ptpn13* | Tyrosine-protein phosphatase non-receptor type 13 | Forward | CCTGATCATGGTGTTCCTGAAGA |
|  |  |  |  | Reverse | TAAGAGTGCCACTCCTTCCG |
| 56 | evm.model.contig_362.2 | *ripk1* | Receptor-interacting serine/threonine-protein kinase 1 | Forward | ACCTTCACTGAGCTTTTTGGA |
|  |  |  |  | Reverse | AGTTTTTGTAGGTGAGCTGGGT |
| 57 | evm.model.contig_7.2 | *septin4* | Septin-4 | Forward | TGAGGACCCCAACCATTGTG |
|  |  |  |  | Reverse | TTCGCCATCTCTTGATCATCA |
| 58 | evm.model.contig_301.3 | *sptan1* | Spectrin alpha chain, non-erythrocytic 1 | Forward | AGAATTGGGAACTGTCATGAGATCA |
|  |  |  |  | Reverse | CAATTGTACCGTTTCCATCAGCA |
| 59 | evm.model.contig_1387.6 | *tnf* | Tumor necrosis factor | Forward | AGTTGCGATATGGAGGGGTG |
|  |  |  |  | Reverse | AGGCACAAATTCAGCTCCGA |
| 60 | evm.model.contig_2341.1 | *tnfrsf1a* | Tumor necrosis factor receptor superfamily member 1A | Forward | TCGGCTTATTTCGGAGGTGT |
|  |  |  |  | Reverse | GCCCCACCATTAGTAGCATTG |
| 61 | evm.model.contig_905.3 | *tuba1b* | Tubulin alpha-1B chain | Forward | AAGTGGGTTAGTGTGGGAATT |
|  |  |  |  | Reverse | TGAAAAAGGTGGACAAGGGT |
| 62 | JN636814.1 | 18s rRNA | 18S ribosomal RNA | Forward | ACCTTCTGGACTGCGCTAAC |
|  |  |  |  | Reverse | CGCAGGTTCACCTACGGAAA |
