# Supplementary figures and images for: Honokiol induces apoptosis-like death in Cryptocaryon irritans Tomont
Source: Parasit Vectors. 2023 Aug 16;16:287. doi: 10.1186/s13071-023-05910-1 (PMC10428556; doi:10.1186/s13071-023-05910-1)

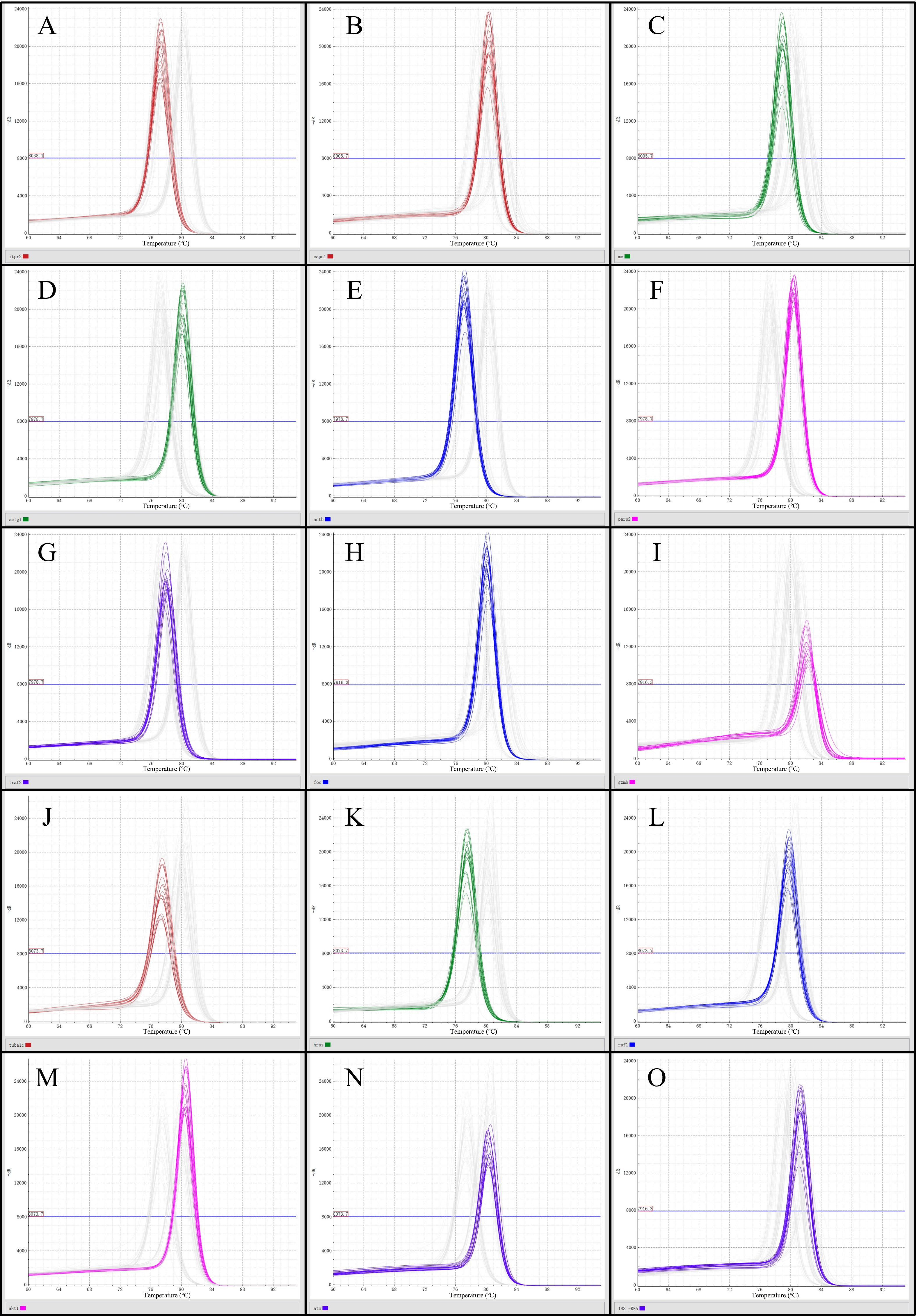

Supplement: Supplementary file 2 — Additional file 2: Fig. S1. The fluorescence quantitative melting curves of the 14 significantly differentially expressed genes. [file 13071_2023_5910_MOESM2_ESM.tiff]
